# Rewiring the Molecular Interplay of CDK4/6 Inhibitors in Lung Cancer: From Cell Cycle Control to Immune Microenvironment Remodeling

**DOI:** 10.3390/ijms27167119

**Published:** 2026-08-08

**Authors:** Yin Ku, Yao Zheng, Yu Ding, Peichuan Zhang, Xiaoqing Wu, Yaohui Chen

**Affiliations:** 1 Department of Thoracic Surgery and Institute of Thoracic Oncology, West China Hospital, Sichuan University, No.37, Guoxue Valley, Wuhou District, Chengdu 610041, China; kuyin@wchscu.cn (Y.K.); zhengyao@wchscu.edu.cn (Y.Z.); dingyu_9966@163.com (Y.D.); zhangpeichuan@wchscu.edu.cn (P.Z.); wuxiaoqing@wchscu.edu.cn (X.W.); 2 Frontiers Science Center for Disease-Related Molecular Network, West China Hospital, Sichuan University, Chengdu 610041, China

**Keywords:** CDK4/6 inhibitors, non-small-cell lung cancer, tumor microenvironment, acquired resistance, biomarkers

## Abstract

Traditional inhibitors of cyclin-dependent kinases 4 and 6 (CDK4/6) have long been characterized as classical antiproliferative agents that induce G1 cell cycle arrest by blocking the phosphorylation of the retinoblastoma protein (Rb). However, recent studies in lung cancer have expanded this paradigm, revealing a functional transition from exclusive tumor suppression to the profound remodeling of the tumor microenvironment (TME) to enhance antitumor immunity. This review systematically outlines the genomic aberrations of the CDK4/6-Rb axis across lung cancer subtypes and dissects its immunomodulatory networks. These encompass the activation of effector T cells, the alleviation of immunosuppression mediated by regulatory T cells (Tregs), and the enhancement of antigen presentation via the Cyclic GMP-AMP synthase-stimulator of interferon genes (cGAS-STING) pathway. Furthermore, we analyze acquired resistance mechanisms, primarily focusing on p21-CDK2 bypass activation mediated by Cyclin E1 gene (*CCNE1*) amplification and tumor protein 53 gene (*TP53*) mutations. We also review clinical investigations combining CDK4/6 inhibitors with targeted therapies against driver genes, as well as immune checkpoint inhibitors in lung cancer. Notably, in the context of lung cancer, these combinatorial strategies have been primarily investigated in the second-line or subsequent settings following progression on standard platinum-based chemotherapy or immunotherapy. Finally, we propose individualized, stratified treatment strategies based on genomic and immunological biomarkers, providing a translational framework for overcoming multidrug resistance and optimizing next-generation combinatorial regimens in lung cancer.

## 1. Introduction

Lung cancer remains the leading cause of cancer-related mortality worldwide, with non-small-cell lung cancer (NSCLC) accounting for approximately 85% of all cases [1,2]. Although targeted therapies against classical driver mutations [e.g., epidermal growth factor receptor (EGFR), anaplastic lymphoma kinase (ALK)] represent the mainstay of treatment for lung adenocarcinoma, and immune checkpoint inhibitors (ICIs) have established the standard of care for lung squamous cell carcinoma, intrinsic and inevitable acquired resistance remains a major barrier limiting clinical benefit [3,4,5,6]. This challenge is particularly evident in lung squamous cell carcinoma (LUSC), which lacks classical actionable targets, and in refractory NSCLC following multiple lines of therapy, underscoring the urgent need for novel therapeutic interventions.

The dysregulation of cell cycle progression is a fundamental hallmark of the initiation and progression of most malignancies [7]. In hormone receptor-positive breast cancer, the development and combinatorial use of highly selective Cyclin-dependent kinases 4 and 6 (CDK4/6) inhibitors have established a substantial clinical precedent [8,9]. Given the high frequency of cell cycle pathway aberrations in the lung cancer genome [e.g., Cyclin-dependent kinase inhibitor 2A (*CDKN2A*) loss, Cyclin D1 gene (*CCND1*) amplification], extrapolating this translational success to lung cancer is supported by a solid molecular pathology rationale [10]. However, due to the heterogeneity of lung cancer driver genes and complex bypass compensatory mechanisms, early attempts to employ these agents as monotherapies in unselected lung cancer cohorts encountered profound clinical limitations, specifically the lack of uniform overall survival (OS) or progression-free survival (PFS) benefits. For instance, retrospective analysis of the Phase III JUNIPER trial (evaluating abemaciclib versus erlotinib in Kirsten rat sarcoma viral oncogene homolog (*KRAS*) -mutant NSCLC) revealed highly divergent, subtype-dependent outcomes. While abemaciclib conferred an OS advantage to the *KRAS*/*STK11* subtype (KL) and a PFS benefit to both KL and *KRAS*/tumor protein *53* gene (*TP53*) subtypes (KP), patients with isolated *KRAS* (K) mutations derived absolutely no OS or PFS benefit. These mixed endpoints explicitly quantify how unstratified administration fails to deliver broad clinical efficacy [11]. These outcomes have prompted a reevaluation of the precise biological significance of the CDK4/6 node in lung cancer.

Crucially, emerging translational evidence indicates that the biological functions of CDK4/6 kinases extend beyond the traditional framework of cell cycle restriction point regulation. They serve as essential mediators in driving epigenetic remodeling and modulating local antitumor immune responses [12,13]. By concurrently inducing antiproliferative arrest and remodeling the tumor microenvironment (TME), this dual mechanism offers a viable therapeutic strategy for overcoming existing immune and targeted therapy resistance in lung cancer.

Accordingly, to provide a robust theoretical foundation for next-generation targeted and immune combination therapies in lung cancer, this review sequentially traces the genomic alteration landscape of the CDK4/6-retinoblastoma (Rb) axis, dissects its immune remodeling mechanisms, evaluates current therapeutic combinatorial strategies, delineates resistance evolution pathways, and ultimately summarizes the biomarkers.

## 2. Physiological Homeostasis and Pathological Dysregulation of the CDK4/6-Rb Axis in Lung Cancer

### 2.1. The Regulatory Network of the Cell Cycle in Normal Lung Tissue

The precise progression of the cell cycle is the cellular foundation for maintaining lung tissue homeostasis and normal physiological functions (e.g., gas exchange). Normal lung epithelial cells in the quiescent phase (G0) are subject to strict growth inhibition [14]. However, upon receiving mitogenic signals from the microenvironment [e.g., epidermal growth factor (EGF), fibroblast growth factor (FGF)], classical intracellular signaling cascades [such as the Mitogen-activated protein kinase (MAPK)/Extracellular signal-regulated kinase (ERK) and Phosphoinositide 3-kinase (PI3K)/Protein kinase B (Akt) pathways] are activated. This activation rapidly upregulates the expression of D-type cyclins (Cyclin D1, D2, and D3) at both the transcriptional and translational levels [15].

Newly synthesized Cyclin D specifically binds to cytoplasmic CDK4/6. Subsequent phosphorylation by CDK-activating kinase (CAK) forms a fully active Cyclin D-CDK4/6 holoenzyme complex [15]. This active complex translocates to the nucleus, targeting and triggering the mono-phosphorylation of Rb. This initial modification induces a conformational change in Rb, leading to its partial dissociation from the E2 factor (E2F) family of transcription factors (e.g., E2F1-3). Subsequently, Cyclin E-CDK2 mediates continuous, multi-site hyperphosphorylation of Rb, disrupting its pocket structure and facilitating the complete release and deoxyribonucleic acid (DNA) binding of E2F [16,17,18,19]. Activated E2F initiates the transcription of key target genes essential for S-phase DNA replication and centrosome duplication [e.g., Cyclin E1 gene *(CCNE1*), Cell Division Cycle 25A (*CDC25A*), Thymidylate synthetase (*TYMS*), Proliferating cell nuclear antigen (*PCNA*)]. These tightly regulated molecular events irreversibly drive lung epithelial cells past the restriction point (R point), marking the transition from G1 to S phase [20].

To prevent aberrant pathological proliferation of the lung epithelium, these positive signaling cascades are subjected to negative feedback regulation by two classical classes of endogenous cyclin-dependent kinase inhibitors (CKIs). The first is the Inhibitors of CDK4 (INK4) family, primarily represented by p16^INK4A^ (encoded by the *CDKN2A* gene). This protein specifically binds to CDK4/6, inducing an allosteric distortion of the kinase catalytic pocket and competitively inhibiting the assembly of Cyclin D with CDK4/6 [15]. In normal lung epithelium under steady-state conditions, p16^INK4A^ expression is generally maintained at low levels [21]. However, under abnormal replication stress (e.g., oncogene activation, telomere dysfunction, or persistent oxidative stress), its expression is rapidly upregulated [20]. This induces irreversible oncogene-induced senescence (OIS) or triggers sustained cell cycle arrest dependent on the G1 checkpoint [20].

The second class is the CDK-interacting protein/Kinase inhibitory protein (Cip/Kip) family, including p21 (encoded by *CDKN1A*) and p27 (encoded by *CDKN1B*). These proteins exhibit a dual regulatory function during cell cycle progression. In the G1 phase, they bind to and directly inhibit the kinase activity of the Cyclin E/A-CDK2 complex, preventing premature transition across the G1/S boundary. Conversely, during early cellular homeostasis, free p21/p27 can act as non-inhibitory scaffold proteins, facilitating and stabilizing the initial assembly and nuclear translocation of Cyclin D-CDK4/6 complexes [22].

In summary, the precise regulation of mitotic rhythms in normal lung epithelial tissue fundamentally relies on the balance between the positive driving forces of Cyclin-CDK complexes and the negative constraints of CKIs. The G1/S checkpoint serves not only as the central hub for regulating proliferation but also as a critical barrier against oncogenic stress. Because this highly complex dynamic equilibrium is sensitive to regulatory signals, irreversible genomic aberrations in core nodes (such as *CDKN2A*) induced by external mutagens can disrupt this barrier, acting as an early molecular driver of malignant transformation in lung epithelial cells. This physiological rationale establishes the foundation for investigating the pathological heterogeneity of this pathway in lung cancer and its role as a key therapeutic target.

### 2.2. Genomic Alteration Heterogeneity of the CDK4/6 Pathway in Lung Cancer

Dysregulation of the CDK4/6-Rb-E2F axis is a near-universal hallmark in the initiation and progression of lung cancer. However, the molecular mechanisms driving the uncoupling of this pathway exhibit substantial heterogeneity across different histological subtypes. Multi-omics analyses from The Cancer Genome Atlas (TCGA) reveal that in NSCLC, the collapse of cell cycle checkpoints is primarily driven by the disruption of upstream negative regulatory networks or the amplification of positive driving elements [5]. In contrast, in small-cell lung cancer (SCLC), genomic aberrations directly eliminate the core downstream substrates of the pathway [10,23].

The loss of upstream negative regulatory elements is a central early oncogenic event in NSCLC. The homozygous deletion of the *CDKN2A* gene or its epigenetic silencing via promoter hypermethylation is the most common mechanism. This genomic event is significantly enriched in LUSC, with an incidence of 30–40%, while being detected in 10–15% of lung adenocarcinomas (LUAD) [10]. The inactivation of *CDKN2A* (encoding the p16^INK4A^ protein) deprives the cell of its primary allosteric inhibitory capacity against CDK4/6 kinase activity. This allows CDK4/6 to maintain a sustained and aberrantly high activation state even under minimal mitogenic ligand stimulation, enabling tumor cells to overcome contact inhibition.

In comparison, focal amplifications of positive driver genes, such as *CCND1* (encoding Cyclin D1), CDK4, or CDK6, are observed in 15–18% of LUSC cases (predominantly driven by 11q13/*CCND1* amplification), compared to 5–10% in LUAD [10,24,25,26]. Mechanistically, in LUAD, these amplification events rarely occur in isolation and are often highly coordinated with mutations in classical upstream driver genes like Kirsten rat sarcoma viral oncogene homolog (*KRAS*). Aberrant MAPK/ERK signaling cascades driven by mutant *KRAS* persistently induce Cyclin D1 at the transcriptional level and prevent its degradation via phosphorylation, thereby stabilizing Cyclin D1 protein expression [27,28]. The convergence of enhanced upstream signaling and core kinase gene amplification maximizes the proliferative potential of malignant cells, conferring a profound oncogene addiction to the CDK4/6 pathway.

Conversely, in SCLC and a subset of NSCLC undergoing lineage plasticity with neuroendocrine differentiation following resistance to targeted therapies (e.g., Epidermal growth factor receptor tyrosine kinase inhibitors (EGFR-TKIs)), genomic alterations bypass upstream regulation to directly target the core cell cycle substrate, the *RB1* gene [29]. Disruptive mutations or biallelic loss of *RB1* [frequently co-occurring with *TP53* mutations] deprive CDK4/6 of its essential physical substrate. For these tumor cells, the G1/S restriction point is not merely dysregulated but entirely abrogated [30].

**Figure 1:**
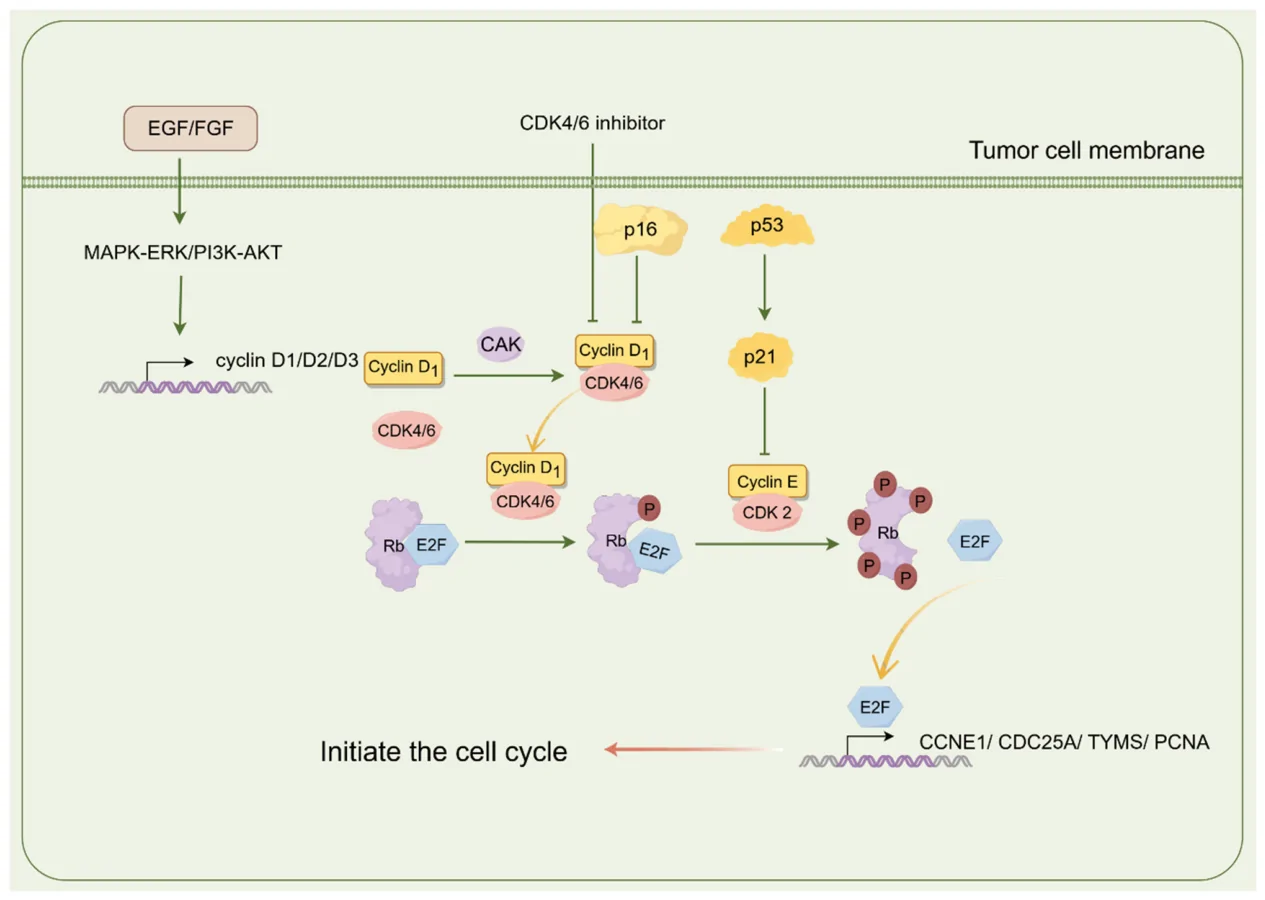
Integrative schematic detailing the regulatory network of the CDK4/6-Rb axis in lung cancer. Upstream mitogenic signals (e.g., EGF/FGF) drive the expression of D-type cyclins via the MAPK/PI3K-AKT pathways. The active Cyclin D-CDK4/6 complex initiates the hypo-phosphorylation of Rb, while the downstream Cyclin E-CDK2 complex promotes Rb hyperphosphorylation, leading to the release of the E2F transcription factor and subsequent transcription of S-phase genes (e.g., *CCNE1*, *CDC25A*). Therapeutic intervention with CDK4/6 inhibitors directly halts this cascade. Concurrently, the p53-p21 axis suppresses Cyclin E-CDK2 activity, providing a dual blockade that prevents Rb hyperphosphorylation, thereby sequestering E2F and inducing robust G1 phase cell cycle arrest. This image was created using Figdraw v2.0.

In conclusion, genomic alterations of the CDK4/6 pathway in lung cancer exhibit distinct subtype-specific patterns, primarily manifesting as upstream p16^INK4A^ loss, aberrant Cyclin D1 amplification, and the basal abrogation of the Rb protein. This molecular heterogeneity characterizes diverse trajectories of malignant evolution in lung cancer and dictates the differential targeted dependency and intrinsic resistance to CDK4/6 inhibitors among patients, establishing a crucial prerequisite for precise molecular stratification in subsequent clinical interventions (Figure 1).

## 3. Discovery and Development of CDK4/6 Inhibitors

The development of drugs targeting cell cycle kinases has faced substantial challenges. Early “pan-CDK inhibitors” (e.g., flavopiridol and roscovitine) failed to precisely distinguish cell cycle-regulating CDKs (e.g., CDK1/2/4/6) from transcription-regulating CDKs (e.g., CDK7/9) within the highly conserved Adenosine triphosphate (ATP)-binding pocket. This lack of selectivity resulted in severe off-target toxicities and intolerable clinical side effects, ultimately limiting their clinical utility [8]. Driven by high-throughput screening and detailed crystallographic analysis, the discovery of selective small-molecule inhibitors precisely targeting CDK4 and CDK6 ultimately achieved a true breakthrough in the druggability of this target [30].

Currently, the most representative highly selective agents in clinical use exhibit distinct molecular and pharmacokinetic profiles. Palbociclib and ribociclib, representing the first generation of highly selective drugs, competitively block the activating modification by CDK-CAK by occupying the ATP catalytic domain of CDK4/6 via non-covalent binding [8]. Both agents exhibit comparable and exceedingly high affinities for CDK4 and CDK6. However, because CDK6 plays an essential role in driving the proliferation of hematopoietic stem and progenitor cells (HSPCs) in the bone marrow, these two drugs frequently induce dose-limiting neutropenia. This necessitates an intermittent dosing schedule (three weeks on, one week off) in clinical practice to allow for the cyclic recovery of bone marrow hematopoiesis [31,32].

In contrast, structural modifications in abemaciclib confer distinct pharmacological advantages. Its kinase inhibitory potency against CDK4 is several times greater than against CDK6 (CDK4 > CDK6) [33]. This target preference significantly reduces hematological toxicity, making it the only CDK4/6 inhibitor suitable for continuous daily oral administration, thereby exerting uninterrupted cell cycle blockade on tumor cells [34]. Furthermore, abemaciclib possesses superior blood–brain barrier (BBB) permeability, achieving effective therapeutic concentrations within the central nervous system [35]. This unique pharmacokinetic property provides a crucial clinical intervention basis for managing the high incidence of brain metastases in lung cancer [34].

Additionally, advancing knowledge of lung cancer pathobiology has catalyzed innovative evolutions in dosing strategies. For instance, trilaciclib, a novel short-acting intravenous formulation, establishes a new mechanism of “myelopreservation and tumor suppression.” By leveraging the physiological divergence wherein normal bone marrow cells depend on the CDK4/6 pathway while SCLC cells do not (due to *RB1* loss), a brief intravenous infusion prior to chemotherapy transiently arrests normal hematopoietic stem cells in the G1 phase. This protects them from cytotoxic agents, achieving precise bone marrow preservation [36]. The intravenous route ensures the highest bioavailability and rapid clearance, synchronizing stem cell arrest precisely with chemotherapy. Unlike oral CDK4/6 inhibitors with variable absorption and prolonged half-lives, which would delay hematopoietic recovery, intravenous trilaciclib acts as a transient “chemical shield” without compromising subsequent marrow regeneration [37].

By continuously evolving from overcoming off-target toxicity to achieving target preference, BBB penetration, and refined myelopreservation, the iterative molecular design of CDK4/6 inhibitors has laid a robust pharmacological foundation for their clinical application in the complex lung cancer microenvironment (Table 1).

## 4. New Perspectives in Immunomodulation of CDK4/6 Inhibitors: From Cell Cycle Arrest to Antitumor Immune Activation

Multiple studies indicate that CDK4/6 inhibitors exert not only classical antiproliferative effects via cell cycle arrest but also play a critical role in modulating TME and enhancing systemic antitumor immune responses [13,45,46,47,48].

### 4.1. Direct Activation of Effector T Cells

CDK4/6 inhibitors can directly act on immune effector cells to enhance their immune functions. Research demonstrates that small-molecule inhibitors of CDK4/6 significantly ameliorate the reduction of monocyte-derived and interstitial macrophages, dendritic cells, γδTCR^+^ T cells, and CD8^+^ effector T cells in peripheral blood caused by other drug treatments [36,49]. Moreover, SCLC patients briefly exposed to trilaciclib during chemotherapy exhibit increased peripheral lymphocyte counts and enhanced T cell activation, indicating that trilaciclib can preserve and augment immune system function in SCLC [49,50].

Mechanistically, the core molecular pathway underlying the immune activation of effector T cells induced by CDK4/6 inhibitors is the CDK6- Nuclear factor of activated T cells (NFAT) axis. NFAT is a critical transcription factor for T cell activation; CDK6 mediates the phosphorylation of NFAT, leading to its inactivation, cytoplasmic retention, and loss of transcriptional regulatory function [51,52]. However, inhibitors such as palbociclib and trilaciclib block CDK6 kinase activity, reducing NFAT phosphorylation and promoting its nuclear translocation. This initiates the transcription of target genes such as IL-2 and IFN-γ, subsequently inducing the antitumor cytotoxicity of effector T cells, increasing their infiltration into the tumor core, and establishing an effective antitumor immune barrier [53].

### 4.2. Alleviation of Immunosuppression

In the process of remodeling antitumor immunity, the alleviation of immunosuppression is equally critical. CDK4 is a key factor in the development and maintenance of regulatory T cells (Tregs), which are the primary mediators of tumor immune tolerance. In the mouse model of lung cancer harboring the *Kras*^LSL-G12D^*Trp53*^fl/fl^ mutation, trilaciclib or palbociclib treatment significantly reduces the number of FOXP3^+^ Tregs and lowers the Treg-to-CD8^+^ cell ratio within intratumoral T cell populations. This attenuates the suppressive effects of Tregs on effector T cells, thereby promoting the immune clearance of tumor cells [13,53].

Mechanistically, CDK4/6 inhibitors act on immunosuppressive Tregs by blocking the CDK4/6-Rb-E2F pathway, which decreases the release of the E2F transcription factor. This directly impairs the transcription and synthesis of DNA methyltransferase 1 (DNMT1) [13]. This impairment consequently relieves the epigenetic repression of *CDKN1A* (encoding the p21 protein) [13,54], leading to substantial p21 expression and irreversible cell cycle arrest in Tregs, thereby fundamentally attenuating their immunosuppressive function [54,55]. In SCLC patients receiving trilaciclib during chemotherapy, the proliferative recovery of Tregs is notably slower than that of CD8^+^ and CD4^+^ T cells. This differential proliferation and recovery result in a decreased ratio of Tregs to effector T cells within the TME, effectively relieving immunosuppression and enhancing immune system function [49,56]. Therefore, CDK4/6 inhibitors exert a direct regulatory effect on immunosuppressive cells, providing a critical mechanistic rationale for the combination of CDK4/6 inhibitors with immune checkpoint therapies.

### 4.3. Enhancement of Tumor Cell Immunogenicity

CDK4/6 inhibitors possess diverse immunomodulatory activities. They can activate functional effector T cells and alleviate immunosuppressive phenotypes within the TME while simultaneously enhancing the immunogenicity of the tumor cells themselves, thereby bidirectionally promoting systemic antitumor immune responses. CDK4/6 inhibitors enhance tumor cell immunogenicity through five distinct mechanisms.

First is inducing viral mimicry responses. Abemaciclib activates the expression of endogenous retroviral elements (ERVs) in tumor cells, leading to elevated levels of double-stranded ribonucleic acid (RNA). This stimulates the production of type III interferons and triggers an antiviral immune response [13].

The second mechanism is enhancing antigen presentation. CDK4/6 inhibitors suppress the CDK4/6-Rb-E2F axis, thereby activating the NF-κB signaling pathway and the type I interferon (IFN I) pathway. This modulation results in the upregulation of major histocompatibility complex class I (MHC-I) on tumor cells and enhances MHC II expression on antigen-presenting cells, such as macrophages and dendritic cells, via potentiation of interferon regulatory factor signaling [57]. Conversely, CDK4/6 inhibition also impedes Programmed death-ligand 1 (PD-L1) degradation, leading to further accumulation of PD-L1 protein on the cell surface. The addition of anti-PD-L1 therapy further augments this immune activation by relieving PD-L1-mediated immune suppression, thereby facilitating enhanced antigen presentation and T cell activation [57,58]. Consequently, the combination of CDK4/6 inhibitors with PD-L1 blockade synergistically enhances both adaptive and innate immune activation [15,59].

The third mechanism is chromatin remodeling. CDK4/6 inhibitors can induce chromatin structural remodeling in tumor cells, activating a highly specific set of enhancers dependent on Activator protein 1 (AP-1) transcriptional activity. These enhancers are predicted to regulate interferon-stimulated genes, potentially promoting the expression of genes associated with interferon signaling pathways [60].

The fourth mechanism is inducing metabolic stress. Drugs targeting CDK4/6 inhibit cell cycle arrest and trigger metabolic stress within tumor cells [61]. In CDK4/6 inhibitor-treated breast cancer cells, intracellular levels of glutamate (a product of glutamine conversion) were increased, and cells continued to uptake nutrients (glucose and glutamine) to supply energy for mTOR-driven biosynthesis, leading to the upregulation of mTOR-mediated glycolytic and oxidative metabolism as well as mitochondrial content [61,62]. This resulted in the accumulation of reactive oxygen species (ROS), which in turn drove the secretion of immunostimulatory chemokines and enhanced the chemotaxis and infiltration of immune cells into the TME [63].

The fifth mechanism is activating the classical Cyclic GMP-AMP synthase-stimulator of interferon genes (cGAS-STING) pathway. CDK4/6 inhibitors (alone or in combination) induce DNA damage in tumor cells, promoting the aberrant formation of micronuclei or cytosolic DNA. This event directly activates the cGAS-STING signaling pathway, triggering type I interferon responses and chemokine secretion. This process enhances tumor immunogenicity and promotes T cell infiltration, providing a theoretical foundation for combination with immune checkpoint therapies [45,64,65].

**Figure 2:**
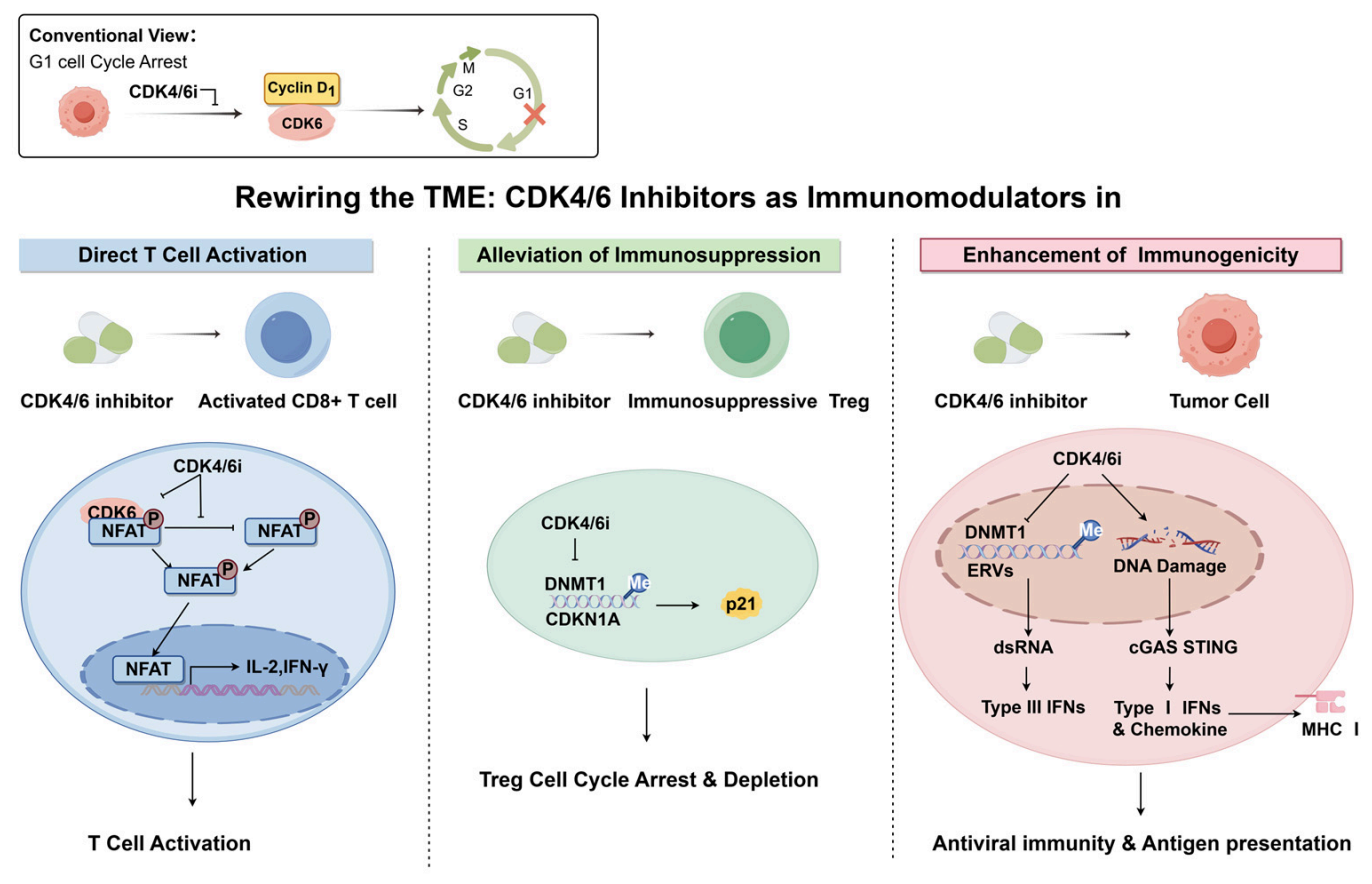
A comprehensive overview of the immunomodulatory mechanisms of CDK4/6 inhibitors within the lung cancer TME. CDK4/6 inhibitors affect antitumor immunity, acting both within cancer cells and on the immune system of the host. In effector T cells, inhibition of CDK4/6 stimulates NFAT transcriptional activity and enhances secretion of IFN-γ and IL2. In regulatory T cells, CDK4/6 inhibitors directly inhibit the cell cycle, thereby blocking cell proliferation. In tumor cells, CDK4/6 inhibitors not only impede the expression of DNMT1, reduce the methylation of ERVs, increase intracellular levels of double-stranded RNA, and enhance type III interferons, but also lead to DNA damage, activate the cGAS-STING pathway, and upregulate the expression of type III interferons, chemokines, and MHC-I. This image was created using Figdraw.

CDK4/6 inhibitors achieve TME remodeling by activating effector T cells, attenuating Treg-mediated immunosuppression, and enhancing antigen presentation via cGAS-STING pathway activation. The conceptual shift from traditional cell cycle arrest to TME remodeling establishes a critical theoretical cornerstone for introducing combination strategies involving cell cycle-targeted agents and immunotherapies in lung cancer (Figure 2).

## 5. Current Application of CDK4/6 Inhibitors in Lung Cancer

### 5.1. Clinical Challenges and Perspectives on Early Monotherapy Exploration

Following the substantial therapeutic success of CDK4/6 inhibitors in hormone receptor-positive breast cancer [8], early clinical investigations attempted to repurpose them as monotherapies for unselected advanced NSCLC across indications. However, this pan-tumor monotherapy strategy encountered significant clinical limitations. For instance, the large-scale Phase III JUNIPER trial evaluated the efficacy of abemaciclib monotherapy versus erlotinib (an epidermal growth factor receptor tyrosine kinase inhibitor) in heavily pretreated advanced NSCLC patients harboring *KRAS* mutations [66]. The results indicated that the overall survival (OS) between the two cohorts did not show a statistically significant improvement [11]. Similarly, palbociclib monotherapy (e.g., the NCT02154490 trial) demonstrated only limited progression-free survival (PFS) benefits in NSCLC cohorts lacking rigorous biomarker selection [67].

These suboptimal clinical outcomes underscore the complex heterogeneity of lung cancer, particularly NSCLC. Unlike breast cancer, which heavily relies on the singular estrogen receptor-induced Cyclin D expression pathway [8], lung cancer cells harbor intricate oncogenic networks [68]. For example, MAPK and PI3K/Akt bypass activation accompanying *EGFR* or *KRAS* mutations can subsequently activate the CDK2/Cyclin E axis or initiate cell cycle-independent bypass pathways to achieve molecular evasion. Therefore, the translational application of CDK4/6 inhibitors in lung cancer cannot simply replicate the intervention paradigms used in breast cancer. To overcome this clinical bottleneck, the therapeutic approach must transition from monotherapy toward combinatorial regimens based on TME remodeling and precise patient stratification based on genomic profiles.

### 5.2. Explorations in Synergistic Efficacy: Multidimensional Combinatorial Strategies with ICIs and TKIs

Combination with ICIs: Given the robust immune-remodeling capacity of CDK4/6 inhibitors, including inducing viral mimicry in tumor cells, activating the cGAS-STING pathway to upregulate MHC-I molecules, and selectively depleting Tregs within the TME, their combination with the Programmed cell death protein 1/Programmed death-ligand 1 (PD-1/PD-L1) blockade has emerged as a major translational focus for overcoming immune resistance in lung cancer [13]. This combination establishes a dual mechanistic synergy: CDK4/6 inhibitors convert poorly immunogenic ‘cold’ tumors into highly lymphocyte-infiltrated ‘hot’ tumors, re-exposing tumor antigens to the immune system, while anti-PD-(L)1 antibodies concomitantly block residual immune evasion signals, fully restoring T cell cytotoxicity. Currently, multiple prospective phase I/II clinical trials (e.g., NCT02779751 evaluating abemaciclib plus pembrolizumab, and NCT03386929 evaluating palbociclib plus avelumab) are actively assessing the therapeutic boundaries of this strategy [69]. *LKB1* mutations lead to primary resistance to immune checkpoint inhibitors, which can significantly affect the effectiveness of immunotherapy in NSCLC patients by influencing TMB and the tumor immune microenvironment [56,70]. However, durable clinical responses were observed in patients with NSCLC who received cdk4/6 inhibitors in combination with immune checkpoint inhibitors [69].

Combination with Driver-Targeted Therapies: In EGFR-mutant NSCLC, the downstream Cyclin D-CDK4/6 axis frequently serves as a common convergence point for various receptor tyrosine kinase (RTK) signaling cascades [15]. During monotherapy with targeted agents (e.g., EGFR-TKIs or Mitogen-activated protein kinase kinase (MEK) inhibitors), tumor cells readily develop acquired resistance via the reactivation of cell cycle bypass pathways [71]. Combining CDK4/6 inhibitors with MEK inhibitors (e.g., NCT03170206 exploring combination with binimetinib) or third-generation EGFR-TKIs (e.g., NCT04545710 evaluating abemaciclib plus osimertinib to reverse resistance) enables a “vertical dual blockade” spanning from upstream membrane receptor signaling to the downstream nuclear mitotic engine. Recent preclinical and preliminary clinical data confirm that this intervention strategy [72], based on linked upstream and downstream targeting, not only induces more profound synergistic apoptosis but also restricts the clonal evolution space of the tumor, effectively delaying or even reversing the emergence of acquired resistance to targeted therapies [16,72].

In summary, whether by remodeling the immune microenvironment to synergize with ICIs or by vertically blocking bypass signaling networks to synergize with TKIs, these multidimensional combinatorial strategies are fundamentally overcoming the limitations of monotherapy. They open promising new clinical avenues for overcoming the complex multidrug resistance barriers in advanced lung cancer.

### 5.3. Genotype-Directed Clinical Benefits and Heterogeneity of Targeted Responses

With the extensive disclosure of data from multiple prospective clinical trials, translational research has clearly delineated a critical paradigm: the targeted sensitivity of lung cancer patients to CDK4/6 inhibitors is strictly governed by their highly heterogeneous baseline genomic defects. In pivotal biomarker-driven umbrella trials (e.g., the Lung-MAP S1400C cohort for NSCLC) [67], patients harboring homozygous *CDKN2A* deletions, focal *CCND1* amplifications, or wild-type *RB1* in the context of *KRAS* mutations have been validated as the primary populations deriving natural benefit from CDK4/6 inhibitors [11]. In these specific molecular subtypes, due to the loss of upstream endogenous negative regulators or the aberrant redundancy of positive stimulatory ligands, malignant proliferation becomes strictly dependent on CDK4/6 kinase activity, thereby exhibiting a high therapeutic response rate to these targeted inhibitors.

Conversely, in subpopulations with loss-of-function mutations or complete deletion of *RB1* (e.g., nearly all SCLC cases, and a subset of targeted therapy-resistant NSCLC undergoing neuroendocrine lineage transformation) [67], or in cohorts with high-level *CCNE1* amplification and *MYC* oncogene overexpression [73], tumor cells have evolutionarily acquired the basal capacity to bypass CDK4/6 nodal control. Under these negative genetic backgrounds, the loss of downstream target proteins or the extensive compensatory activation of CDK2 renders the cell cycle restriction point entirely ineffective. Consequently, both CDK4/6 inhibitor monotherapy and traditional combination regimens yield minimal to no clinical benefit [66,67]. However, this intrinsic resistance also provides a therapeutic window for the “myelopreservation and tumor suppression” mechanism. As demonstrated by multiple SCLC-focused trials, such as NCT02499770 and NCT07473128, trilaciclib is being widely applied for bone marrow protection prior to chemotherapy [36].

Therefore, a paradigm shift from indiscriminate pan-tumor administration to molecularly guided intervention is imperative. In future clinical development involving CDK4/6 inhibitors, implementing baseline genomic profiling via large-panel next-generation sequencing (NGS) is critical. This profiling approach is expected to achieve a minimum sequencing depth of 500× for tissue biopsies to reliably capture low-allele-frequency mutations, homozygous deletions (e.g., *CDKN2A*), and focal copy number amplifications (e.g., *CCND1* or *CCNE1*) [74,75]. For precise molecular stratification is no longer merely an auxiliary tool for optimizing efficacy but rather a fundamental prerequisite determining the success or failure of these targeted therapies [5] (Table 2).

Translational evidence demonstrates that baseline genomic heterogeneity strictly dictates therapeutic responses to CDK4/6 inhibitors, with *CDKN2A*/*CCND1* aberrations conferring targeted sensitivity and *RB1*/*CCNE1* alterations driving intrinsic resistance. Consequently, transitioning from unselected pan-tumor administration to NGS-based molecular stratification is now an absolute prerequisite for optimizing immediate clinical decision-making.

## 6. Mechanisms of Resistance and Overcoming Strategies

### 6.1. Elucidating the Roots of Resistance: From RB1 Loss to Compensatory CDK2 Activation

In SCLC, homozygous inactivation of *RB1* is observed in 94% of cases [45,76]. Because the antiproliferative effects of CDK4/6 inhibitors (e.g., palbociclib, abemaciclib) are entirely dependent on the presence of functional, intact Rb protein within the target cell to bind E2F, this genomic feature dictates intrinsic resistance to CDK4/6 inhibitor [77]. In this scenario, resistance is an innate baseline characteristic driven by the complete absence of the drug’s essential downstream effector.

In stark contrast to this primary evasion, NSCLC patients with specific genomic profiles (e.g., *CDKN2A* deletion) initially demonstrate objective responses to CDK4/6 inhibitors [78]. However, tumor cells inevitably develop acquired resistance under prolonged pharmacological selection pressure [79]. In-depth investigations into these evasion pathways reveal that the most prevalent and critical mechanism is cell cycle rewiring centrally driven by *CCNE1* (encoding Cyclin E1) amplification, compensatory CDK2 activation, and the p21-CDK2 bypass axis [55,80].

*CCNE1* Amplification and CDK2-Driven Activation: The *CCNE1* gene was reported to be highly expressed in LUAD and LUSC tissues, which was also correlated with LUAD and LUSC patients’ overall and disease-specific survival [81,82]. Under normal physiological conditions, the G1-to-S transition requires the sequential activity of Cyclin D-CDK4/6 and Cyclin E-CDK2. However, tumor cells exposed to long-term CDK4/6 inhibition selectively amplify *CCNE1* [83]. The overexpressed Cyclin E1 robustly binds to and activates CDK2. This highly active Cyclin E-CDK2 complex substitutes for CDK4/6 to phosphorylate the Rb protein at multiple sites (S807/811, T373, S608) [84]. Consequently, tumor cells bypass the pharmacologically blocked CDK4/6-Rb-dependent G1/S checkpoint, forcibly releasing the E2F transcription factor and initiating DNA replication. This overcomes cell cycle arrest and establishes a compensatory pathway that drives continuous cell cycle progression, ultimately culminating in acquired resistance [73]. Concurrently, the compensatory activation of the CDK2-Cyclin E axis can further alleviate transforming growth factor beta (TGF-β)-mediated growth inhibition through crosstalk with non-canonical pathways, such as Smad3, conferring additional proliferative advantages to the tumor cells [85].

*TP53* Mutation-Mediated p21-CDK2 Bypass Evasion: This resistance bypass pathway is particularly prominent in LUSC, where it is highly coupled with prevalent disruptive *TP53* mutations. Under normal physiological conditions, wild-type p53 transcriptionally activates p21 (encoded by *CDKN1A*) to establish a critical p21-CDK2 negative feedback loop. This loop suppresses the Cyclin E/CDK2 complex, prevents Rb phosphorylation, and ultimately arrests cells in the G1 phase [86].

However, mutant p53 loses this essential transcriptional activation capacity. The direct consequence is a profound downregulation of p21, which completely eliminates the negative feedback inhibition on CDK2. Freed from p21 control, CDK2 remains aberrantly hyperactive. Therefore, even if exogenous inhibitors completely blockade CDK4/6, this unrestricted CDK2 continuously drives cell cycle progression. Ultimately, this TP53-p21-CDK2 evasion axis allows tumor cells to bypass the G1/S checkpoint, leading to sustained proliferation and acquired clinical resistance [87].

**Table 2:** Clinical trials of CDK4/6 inhibitors as monotherapy and combinatorial regimens in lung cancer.

Intervention	NCT Number	Inclusion Criteria	Study Type	Clinical Phase	Recruitment Status	References
Palbociclib	NCT03170206	*KRAS* Mutant NSCLC	Interventional	Phase I	Completed	Data on file
	NCT03386929	NSCLC Metastatic, NSCLC Stage IIIB	Interventional	Phase I	Terminated	[69]
	NCT02664935	NSCLC	Interventional	Phase II	Completed	Data on file
	NCT02785939	Squamous Cell Lung Cancer Stage IV	Interventional	Phase II	Completed	Data on file
	NCT02154490	Squamous Cell Lung Stage IV Carcinoma AJCC v7	Observational	Phase II/III	Completed	[67]
Ribociclib	NCT03333343	EGFR-mutant NSCLC	Interventional	Phase Ib	Active	Ongoing
	NCT02292550	NSCLC	Interventional	Phase Ib/II	Completed	Data on file
	NCT02411591	NSCLC Stage IV	Interventional	Phase Ib	Completed	[88]
	NCT02079636	NSCLC Stage IV	Interventional	Phase Ib	Completed.	[89]
	NCT02779751	NSCLC, Breast Cancer	Interventional	Phase Ib	Active	Ongoing
	NCT02450539	NSCLC Stage IV	Interventional	Phase II	Completed	Data on file
	NCT04010357	*RB* Wild-type Extensive SCLC, Large Cell Neuroendocrine Lung Cancer, Extrapulmonary Small-Cell Cancers	Interventional	Phase II	Completed	Data on file
	NCT02308020	Breast Cancer, NSCLC, Melanoma, Brain Metastases	Interventional	Phase II	Completed	[90]
	NCT04545710	NSCLC With EGFR Activating Mutations With Osimertinib Resistance	Interventional	Phase II	Active	Ongoing
	NCT02152631	NSCLC Stage IV With a Detectable *KRAS* Mutation	Interventional	Phase III	Active	[66]
Trilaciclib	NCT02499770	SCLC	Interventional	Phase Ib/IIa	Completed	[36]
	NCT06992739	NSCLC	Interventional	Phase II	Recruiting	Ongoing
	NCT06929936	Locally Advanced or Metastatic NSCLC	Interventional	Phase II	Recruiting	Ongoing
	NCT06490081	Myelosuppression in Patients With Limited-stage SCLC	Interventional	Phase II	Recruiting	Ongoing
	NCT07473128	Limited-Stage SCLC	Interventional	Phase III	Recruiting	Ongoing
	NCT05874401	Extensive Stage Small-Cell Lung Cancer	Interventional	Phase IV	Recruiting	Ongoing

NSCLC, Non-small-cell lung cancer; KRAS, Kirsten rat sarcoma viral oncogene homolog; SCLC, Small-cell lung cancer; *RB*, Retinoblastoma gene; EGFR, Epidermal growth factor receptor.

### 6.2. Innovative Strategies to Reverse Bypass Resistance: Clinical Exploration of Next-Generation Inhibitors

To overcome the aforementioned resistance evasion bypasses centered on CDK2 activation, the development of next-generation CDK inhibitors or the design of rational combinatorial regimens has become a clinical translation priority for overcoming acquired resistance. Currently, strategies to resolve this challenge primarily focus on the following two complementary approaches:

Combination with Selective CDK2 Inhibitors: To address CDK2 activation driven by *CCNE1* amplification or *TP53* mutation/p21 loss, clinical efforts are actively advancing the combination of existing CDK4/6 inhibitors with novel small-molecule selective CDK2 inhibitors (e.g., PF-07104091, BLU-222, INCB123667) [86,91,92]. Preclinical studies utilizing lung cancer organoids and patient-derived xenograft models demonstrate that this “dual blockade” strategy can fundamentally impede the two core kinase pathways governing the G1-S transition [93]. This approach resensitizes tumor cells lacking p21 protection to cell cycle arrest or apoptosis and effectively reverses tumor nodules that have already developed resistance to abemaciclib [93].

Development of Next-Generation Multi-Target CDK2/4/6 Inhibitors: Another major exploration involves developing broad-spectrum, multi-target cell cycle inhibitors (e.g., NUV-422, PF-07220060) as single agents capable of simultaneously and equally targeting CDK2, CDK4, and CDK6 [94,95]. These next-generation drugs leverage the high structural conservation of the ATP-binding catalytic pockets within this kinase family, aiming to achieve simultaneous multi-target intervention in first-line clinical therapy. The core developmental rationale is to pre-emptively block the potential kinase pathways tumor cells use for evasion: namely, upstream CDK4/6 activation driven by *CDKN2A* loss, and downstream compensatory escape via CDK2 under prolonged pharmacological selection pressure [87].

In summary, the evolution of resistance to CDK4/6 inhibitors in lung cancer is fundamentally a system-level rewiring of kinase networks driven by extreme pharmacological selection pressure. Beyond the primary resistance barrier mediated by the loss of the *RB1* substrate, compensatory CDK2 activation constitutes the core evasion mechanism by which NSCLC overcomes G1/S blockade, a process centrally driven by *CCNE1* amplification and defects in the p53-p21 signaling axis. Confronted with this complex kinase compensation dilemma, current clinical translation strategies have explicitly shifted from targeting single nodes to achieving comprehensive blockade. Whether utilizing selective CDK2 inhibitors to achieve a dual pathway blockade or relying on next-generation multi-target CDK2/4/6 inhibitors for basal suppression, these approaches aim to dismantle the redundant cell cycle evasion capabilities of tumor cells, providing a highly prospective strategic direction for finally overcoming the resistance bottlenecks in targeted therapy.

## 7. Biomarker Screening in the Context of Precision Medicine

### 7.1. Positive Predictive Factors Driving Targeted Sensitivity

The systematic exploration of positive predictive biomarkers for tumor response to CDK4/6 inhibitors is a core research focus for optimizing individualized therapy and precisely identifying cohorts likely to benefit. Currently, widely validated core positive predictive biomarkers primarily include the functional integrity of the Rb protein, CDK4 amplification, the deletion or mutational inactivation of *CDKN2A*, and the amplification of the cyclin-encoding gene *CCND1*.

#### 7.1.1. Functional Integrity of the Rb Protein as an Essential Prerequisite

The core antiproliferative mechanism of classical CDK4/6 inhibitors generally relies on blocking CDK4/6-mediated phosphorylation of the Rb, thereby inducing G1-phase arrest in target cells. Therefore, the presence of structurally normal and functionally intact Rb protein within the cell is a prerequisite for the antitumor efficacy of CDK4/6 inhibitors [44]. Only 6% of SCLC patients exhibit normal *RB1* functional expression, suggesting a limited potential for CDK4/6 inhibitor monotherapy [76]. Nevertheless, the novel CDK4/6 inhibitor G1T28 (trilaciclib) offers a distinct therapeutic advantage by transiently and reversibly modulating the proliferation of normal HSPCs, which rely on the CDK4/6-Rb pathway, thereby alleviating chemotherapy-induced myelosuppression [44,96]. Thus, assessing the functional status of the Rb protein in tumor tissues serves as a critical clinical indicator for deploying CDK4/6 inhibitors. Tumors with functional *RB1* may benefit from CDK4/6 inhibitors, whereas for *RB1*-deficient tumors, CDK4/6 inhibitor monotherapy is not recommended.

In SCLC, the therapeutic window for CDK4/6 inhibitors is not derived from differential sensitivity of tumor cells to the drug itself, but rather from the selective protection of normal bone marrow against chemotherapy toxicity. Because SCLC cells typically harbor *RB1* loss-of-function mutations, they are insensitive to CDK4/6-mediated cell cycle arrest [44]. Consequently, the optimal strategy to maximize the therapeutic window is to prioritize a combination regimen of CDK4/6 inhibitors with cytotoxic chemotherapeutic agents (such as Topotecan used in SCLC) [44]. The CDK4/6 inhibitors shield normal hematopoietic cells without protecting the tumor, creating a unique uncoupling of host protection and antitumor activity that expands the therapeutic index of the combination regimen.

#### 7.1.2. High Therapeutic Response in Tumors with CDK4 Amplification

CDK4 amplification is present in approximately 10% of treatment-naive metastatic NSCLC patients harboring *EGFR* mutations [97]. Based on established targeted therapy paradigms in liposarcoma, *CDK4* amplification has been validated as an effective biomarker for CDK4/6 inhibitor sensitivity. The amplification and overexpression of CDK4 drive proliferation in liposarcoma, and the CDK4/6 inhibitor palbociclib has demonstrated significant antitumor activity in this malignancy [98]. By extension, NSCLC subpopulations harboring *CDK4* amplifications may exhibit similar sensitivity to CDK4/6 inhibitors. Further investigations into the genomic landscape of NSCLC patients with varying responses to EGFR-TKIs reveal significant differences in cell cycle pathways. Specifically, amplification of CDK4/6 was significant in de novo resistance, indicating that aberrant CDK4/6 amplification is a mechanism underlying primary TKI resistance [99,100]. In preclinical models, the combination of CDK4/6 inhibitors and EGFR inhibitors exerts synergistic antitumor effects in *EGFR*-mutant LUAD cells. The co-treatment with CDK4/6 and EGFR inhibitors can restore proliferative arrest, induce tumor cell apoptosis, and decrease genomic instability by mitigating replication stress and DNA damage [101]. Clinical case reports also provide direct evidence showing that lung cancer patients with concurrent *EGFR* mutations and CDK4 amplifications derive significant benefit from combined targeted therapies involving CDK4/6 inhibitors [102], indirectly corroborating the sensitivity of *CDK4*-amplified lung cancer subsets to cell cycle-targeted agents. These findings suggest that *CDK4* amplification can serve as an effective predictor of sensitivity to CDK4/6 inhibitors in lung cancer patients.

#### 7.1.3. High Frequency of *CDKN2A* Inactivation in Lung Cancer and Its Predictive Implications

The p16 protein (a member of the INK4 family encoded by the *CDKN2A* gene) functions as an endogenous inhibitor of CDK4/6. It specifically binds to CDK4/6 kinase monomers, competitively blocking their assembly with Cyclin D, thereby basally inhibiting the catalytic activity of the holoenzyme complex [103]. Macroscopic sequencing analyses from large-scale cancer genomic databases (e.g., TCGA) clearly demonstrate the high-frequency distribution of *CDKN2A* inactivation in lung cancer. In LUAD, the incidence of homozygous deletions of *CDKN2A* and *CDKN2B* reaches 19.1% and 5.7%, respectively [104,105]. In LUSC, which typically lacks classical driver mutations, the overall inactivation rate of *CDKN2A* is as high as approximately 72% [25]. Overall, the incidence of *CDKN2A* homozygous deletion in NSCLC is maintained at approximately 21% [106]. Studies indicate that LUSC cell lines and NSCLC subpopulations with *CDKN2A* deletions exhibit aberrant CDK4/6 kinase activation [93,107,108] and demonstrate sensitivity to the CDK4/6 inhibitor palbociclib, with good tolerability observed following monotherapy [109,110]. This mechanism establishes *CDKN2A* deletion as a positive biomarker for stratifying patient cohorts likely to benefit from CDK4/6 inhibitors.

#### 7.1.4. Aberrant CDK4/6 Kinase Activation Mediated by *CCND1* Amplification Confers Drug Sensitivity

Tumors harboring *CCND1* gene amplifications are highly dependent on the CDK4/6-Rb proliferative pathway and therefore exhibit intrinsic sensitivity to CDK4/6 inhibitors [15]. In experimental models utilizing *CCND1*-amplified or overexpressing NSCLC cells, the CDK4/6 inhibitor abemaciclib effectively blocked cell proliferation, validating the correlation between Cyclin D1 amplification and drug sensitivity [111]. Furthermore, data from Phase Ib/II clinical trials involving solid tumors with CDK4/6 pathway activation (including cohorts with *CCND1* amplification) demonstrate that the *CCND1*-amplified subpopulation exhibits an improved response to the combination of abemaciclib and paclitaxel [112], reiterating that aberrant Cyclin D1 amplification is a positive predictor of clinical benefit from CDK4/6 inhibitors [113].

### 7.2. Negative Predictive Factors Indicating Primary and Acquired Resistance

Negative predictive factors indicate a high probability of primary or acquired resistance to CDK4/6 inhibitors. Typical high-risk features include *RB1* functional loss, *CCNE1* amplification, and epigenetic silencing induced by Serine/threonine kinase 11/Liver kinase B1 (*STK11*/*LKB1*) deletion. The precise identification of these high-risk profiles holds significant clinical value for avoiding ineffective treatments and optimizing patient stratification.

#### 7.2.1. The Root of Primary Resistance: Decisive Abrogation of Efficacy by *RB1* Functional Loss

As previously noted, *RB1* loss fundamentally disrupts the critical protective mechanisms of the cell cycle restriction point [114]. Consequently, the functional integrity of the Rb protein is a prerequisite for the classical antitumor effects of CDK4/6 inhibitors [44]. Acquired resistance to CDK4/6 inhibitors in patients may be attributed to genetic alterations in the *RB1* gene [114,115]. Clinical evidence further supports the value of *RB1* loss as a resistance biomarker. In the vast majority of SCLC cases, the absence of Rb protein expression resulting from *RB1* mutations renders tumors insensitive to CDK4/6 inhibitor monotherapy [45,76]. In *RB1*-deficient SCLC cell lines, CDK4/6 inhibition induces no changes in the cell cycle, and the proportion of cells in the G1 phase remains unaltered, confirming that *RB1*-deficient cells possess intrinsic resistance to CDK4/6 inhibition [44]. Conversely, Rb-proficient SCLC cell lines exhibit growth-inhibitory sensitivity to CDK4/6 blockade, and clinical reports have described an SCLC patient with positive Rb expression achieving significant tumor regression from abemaciclib monotherapy [45,116].

#### 7.2.2. Bypass of the G1-S Checkpoint: Acquired Resistance Mediated by *CCNE1* Amplification

Single-cell analyses reveal that high Cyclin E1-CDK2 activity can drive the G1-to-S transition independently of CDK4/6 [84]. Cells with high CDK2 activity can maintain Rb phosphorylation and complete S-phase entry even in the presence of high concentrations of palbociclib [84]. In the PALOMA-3 trial, the median PFS for patients with high *CCNE1* mRNA expression treated with palbociclib was only 7.6 months, compared to 14.1 months in the low-expression cohort, indicating a significant correlation between high *CCNE1* expression and resistance to CDK4/6 inhibitors [117]. Additionally, the Palbociclib clinical trial independently validated the reduced antiproliferative activity of palbociclib in the context of high *CCNE1* mRNA levels in Metastatic Breast Cancer [117]. Therefore, *CCNE1* amplification, which activates CDK2 to maintain an Rb phosphorylation bypass, constitutes a core compensatory mechanism for acquired resistance to CDK4/6 inhibitors. This finding also suggests that *CCNE1* expression profiling can be utilized to stratify and identify patients who might benefit from combinatorial regimens involving CDK2 inhibitors, while targeting downstream effectors like CDK2 or polo-like kinase 1 (PLK1) holds promise for reversing this type of resistance [85,118].

#### 7.2.3. Persistent “Cold” Immune Microenvironment: Epigenetic Silencing Due to *STK11/LKB1* Deletion

The deletion of *STK11*/*LKB1* represses the cGAS-STING pathway via epigenetic modification, silencing STING expression [119]. This aberration occurs at a highly elevated frequency in *KRAS*-mutant lung cancers [119]. Tumors harboring co-mutations in both genes universally exhibit downregulation of STING at both the transcriptional and protein levels, impairing type I interferon secretion and compromising innate immune pathway functions, ultimately establishing an immune-evasive phenotype [120]. Inactivating *STK11* mutations are present in broad NSCLC populations, and the vast majority of these mutant cases are accompanied by concurrent *KRAS* mutations. This specific molecular subtype presents a “cold” tumor microenvironment characterized by sustained suppression of the innate immune pathway (cGAS-STING), forming an immunosuppressive niche, and representing a highly prevalent, high-risk molecular subtype in lung cancer [121,122,123]. This phenotype not only confers resistance to ICI monotherapy but also attenuates the clinical benefits of combinatorial therapies involving CDK4/6 inhibitors and immunotherapy, resulting in reduced regimen efficacy [56]. Thus, *STK11*/*LKB1* loss serves as a critical negative biomarker.

## 8. Future Perspectives and Conclusions

In summary, the clinical role of CDK4/6 inhibitors in lung cancer therapy has transcended the traditional paradigm of mere “cytostatic agents” that induce cell cycle arrest. They have evolved into critical “immunomodulatory hubs” capable of profoundly remodeling the tumor immune microenvironment and robustly activating host antitumor immune networks [13]. This dual functionality offers a highly promising therapeutic avenue for patients with refractory lung cancer lacking classical driver mutations, as well as those facing bottlenecks due to targeted therapy resistance.

Facing the highly heterogeneous landscape of lung cancer, successfully advancing these next-generation combinatorial regimens into definitive Phase III clinical trials necessitates three exact translational steps.

First, early-phase trials must leverage large-cohort sequencing and advanced translational models (such as patient-derived xenografts) to precisely define synergistic boundaries and establish stringent, biomarker-driven inclusion criteria across diverse genomic backgrounds (e.g., homozygous *CDKN2A* deletion or *STK11*/*LKB1* mutation) [123].

Second, accelerated Phase I/II clinical evaluations of highly selective next-generation CDK2 or broad-spectrum multi-target CDK inhibitors are required to establish optimal dosing schedules and mitigate overlapping toxicities while simultaneously suppressing compensatory resistance networks (e.g., *CCNE1* amplification and *TP53* mutation-mediated p21-CDK2 bypass) [93,124].

Finally, before initiating Phase III designs, exploratory NGS-based molecular stratification and multiplexed TME phenotypic profiling must be standardized into routinely applicable, clinically validated companion diagnostic assays. The standard for TME phenotype analysis comprises three dimensions: effector immune cell function (IL-2, IFN-γ, and NFAT), relief of immunosuppression (Treg/CD8+, DNMT1-p21), and enhanced tumor immunogenicity (ERV, MHC-I/II, cGAS-STING, ROS). Multiplex immunohistochemistry, flow cytometry, and single-cell RNA-seq are recommended as combined assessment modalities for achieving precise classification of the TME transition from an immunosuppressive to an immune-supportive state [13,125]. Once these precise stratification criteria and safety profiles are solidified, individualized therapeutic regimens seamlessly integrating cell cycle-targeted agents with immunotherapies will unequivocally occupy a central position in the future paradigm of precision medicine for lung cancer.

Facing the highly heterogeneous landscape of lung cancer, future basic research and clinical translation should converge on three core directions: First, leveraging large-cohort sequencing and advanced translational models, such as patient-derived xenografts, is necessary to precisely map the pharmacodynamic profiles and synergistic boundaries of CDK4/6 inhibitors across diverse genomic backgrounds (e.g., homozygous *CDKN2A* deletion or *STK11*/*LKB1* mutation) [123]. Second, the accelerated clinical evaluation of highly selective next-generation CDK2 inhibitors or broad-spectrum multi-target CDK inhibitors is required to address the compensatory resistance networks centrally driven by *CCNE1* amplification and *TP53* mutation-mediated p21-CDK2 bypass activation [93], aiming to achieve basal blockade and comprehensive suppression of cell cycle evasion pathways [124]. Finally, with the maturation of NGS-based molecular stratification and multiplexed TME phenotypic profiling, individualized therapeutic regimens seamlessly integrating cell cycle-targeted agents with immunotherapies will unequivocally occupy a central position in the future paradigm of precision medicine for lung cancer.

## Figures and Tables

**Table 1:** Historical development of CDK4/6 inhibitors.

Representative Drug(s)	Core Target(s)	Chemical Formula and Structural/Pharmacokinetic Characteristics	Developer	FDA Approval Years	References
Flavopiridol (Alvocidib), Roscovitine	Pan-CDK (CDK1/2/4/6 and transcriptional CDK7/9)	Lack of selectivity, unable to precisely distinguish highly conserved ATP-binding pockets; extreme toxicity.	Sanofi, Cyclacel (Early pan-target representatives)		[8,38]
Palbociclib	CDK4 = CDK6	Non-covalent, competitive binding to the ATP catalytic cleft; long half-life; induces dose-limiting myelosuppression, requiring a “3 weeks on, 1 week off” intermittent dosing schedule.	Pfizer	24 June 2026	[39,40]
Ribociclib	CDK4 = CDK6	Pharmacological mechanism is highly similar to palbociclib; significantly inhibits HSPC proliferation, necessitating intermittent dosing.	Novartis	13 March 2017	[41,42]
Abemaciclib	CDK4 > CDK6	Unique fluorine side-chain modification reduces hematological toxicity, enabling continuous daily oral administration; high lipophilicity confers excellent BBB penetrability.	Eli Lilly	12 October 2021	[34,43]
Trilaciclib	CDK4/6	Short-acting intravenous formulation, administered via brief infusion prior to chemotherapy; leverages the pathway dependency divergence between SCLC (*RB1*-deficient) and normal cells to achieve precise myelopreservation.	G1 Therapeutics/Simcere	12 February 2021	[36,44]

CDK, Cyclin-dependent kinases; ATP, Adenosine Triphosphate; HSPC, Hematopoietic Stem and Progenitor Cells; BBB, blood–brain barrier; SCLC, Small-cell lung cancer; *RB1*, Retinoblastoma 1 gene.

## Data Availability

No new data were created or analyzed in this study. Data sharing is not applicable to this article.

## Abbreviations

The following abbreviations are used in this manuscript:

Abbreviation	English Full Name
Akt	Protein kinase B
ALK	Anaplastic lymphoma kinase
AP-1	Activator protein 1
ATP	Adenosine triphosphate
BBB	Blood–brain barrier
CAK	CDK-activating kinase
*CCNE1*/*CCND1*	Cyclin E1/D1 gene
CDC25A	Cell division cycle 25A
CDK4/6	Cyclin-dependent kinases 4 and 6
CDKN2A/CDKN1A/CDKN1B	Cyclin-dependent kinase inhibitor 2A/1A/1B
cGAS-STING	Cyclic GMP-AMP synthase-stimulator of interferon genes
Cip/Kip	CDK-interacting protein/Kinase inhibitory protein
CKIs	Cyclin-dependent kinase inhibitors
DNA/RNA	Deoxyribonucleic acid/Ribonucleic acid
DNMT1	DNA methyltransferase 1
E2F	E2 factor
EGF	Epidermal growth factor
EGFR	Epidermal growth factor receptor
ERK	Extracellular signal-regulated kinase
ERVs	Endogenous retroviral elements
FGF	Fibroblast growth factor
HSPCs/HSPC	Hematopoietic stem and progenitor cells
ICIs/ICI	Immune checkpoint inhibitors
INK4	Inhibitors of CDK4
KRAS	Kirsten rat sarcoma viral oncogene homolog
LUAD	Lung adenocarcinoma
LUSC	Lung squamous cell carcinoma
MAPK	Mitogen-activated protein kinase
MEK	Mitogen-activated protein kinase kinase
MHC-I/MHC-II	Major histocompatibility complex class I/II
NFAT	Nuclear factor of activated T cells
NGS	Next-generation sequencing
NSCLC	Non-small-cell lung cancer
OIS	Oncogene-induced senescence
OS	Overall survival
PCNA	Proliferating cell nuclear antigen
PD-1/PD-L1	Programmed cell death protein 1/Programmed death-ligand 1
PFS	Progression-free survival
PI3K	Phosphoinositide 3-kinase
PLK1	Polo-like kinase 1
Rb	Retinoblastoma protein
*RB1*	Retinoblastoma 1 gene
RTK	Receptor tyrosine kinase
SCLC	Small-cell lung cancer
STK11/LKB1	Serine/threonine kinase 11/Liver kinase B1
TCGA	The Cancer Genome Atlas
TGF-β	Transforming growth factor beta
TKIs/EGFR-TKI	Tyrosine kinase inhibitors/Epidermal growth factor receptor tyrosine kinase inhibitor
TME	Tumor microenvironment
*TP53*/p53	Tumor protein 53
Tregs/Treg	Regulatory T cells
TYMS	Thymidylate synthetase
